# Supplementary material for: Patterns of Intron Gain and Loss in Fungi
Source: PLoS Biol. 2004 Nov 30;2(12):e422. doi: 10.1371/journal.pbio.0020422 (PMC532390; doi:10.1371/journal.pbio.0020422)
Supplement: Table S1 — Also available at http://genes.mit.edu/NielsenEtAl/. (4.3 MB ZIP). [file pbio.0020422.st001.zip › NielsenEtAl/html/458.html]

AN9148.1.NCU02797.1.MG01631.1.FG00524.1


```
 CLUSTAL W (1.82) Multiple Sequence Alignments - Introns Inserted


Sequence 1: NCU02797.1	546 aa
Sequence 2: MG01631.1	529 aa
Sequence 3: FG00524.1	513 aa
Sequence 4: AN9148.1	566 aa
Alignment Length: 581 aa
Number Identitical Residues: 379 aa
Alignment Score (without introns) 15915


MG01631.1 	MASNVAKAVKSALPSHLKPESAETKGDSEFAPRHHGKTQSHM0A----------------
NCU02797.1	----MAQAIRSALPDRLMP----SNGDEEFAKRHHGKTRSHM~VSTILSLCHHPSN----
FG00524.1 	----MANIIKSALPTHLKP----NTGDEQGNERRHGKTRSHM0A----------------
AN9148.1  	-------MATKDLPTHLSS-------NTGFG-KHHGKTQSHM~VSSVLCLCVDSFSGFRG
          	          . ** :* .       :     ::****:*** .::  . .  . ..  .

MG01631.1 	---------~--------------------------~-FENASTNIAAAQMRNALTALAE
NCU02797.1	---------~--------------KMTVQEELTHRQ~AFENTSTSIAAAQMRNALTKLAE
FG00524.1 	---------~--------------------------~-FENTSTNVAAAQMRNALTNLAE
AN9148.1  	AWKAPPAMT2DASGYAICSQDVMSQTALQLCSLGTQ0AFENASTSVAASQMRNALNALAE
          	: .:..: : .::. : .:..  :. : .      . :***:**.:**:******. ***

MG01631.1 	T~VTDEKQKN0LFETEMDNFFALFRRFVNDKAKGSTV2DWDRIAPPAQGQVVDYDELANS
NCU02797.1	T~VKDPDQKK~LFETEMDNFFSLFRRYLNDKAKGNEV2NWDKIAPPAPHQIVDYESLANN
FG00524.1 	T0--------~LFETEMDNFFALFRRYLNDKAKGNAV2DWDRIAPPAQGQVVDYDDLANT
AN9148.1  	T~VPDPNERK~RFEAEMDNFFALFRRFLNDKAKGNVV~NWDRIAPPQPSQVVNYDDIGKE
          	*   . .. .  **:******:****::******. * :**:****   *:*:*:.:.: 

MG01631.1 	ESVEFLSKLAVLKLNGGLGTSMGCVGPKSVIEVRDGMSFLDLSVRQVEHLNRTYGTNVPF
NCU02797.1	NSVDFLNKLAVLKLNGGLGTSMGCVGPKSVIEVRDGMSFLDMSVRQVEHLNRTYGSNVPI
FG00524.1 	ESVQFLNKLAVLKLNGGLGTSMGCVGPKSVIEVRDGMSFLDLSVRQIEYLNRTYDVNVPF
AN9148.1  	SSVEFLNKLAVVKLNGGLGTSMGCVGPKSVIEVREGMSFLDLSVRQIEHLNRTYNVNVPF
          	.**:**.****:**********************:******:****:*:*****. ***:

MG01631.1 	ILMNSFNTDEDTASIIKKYEGHNVDIMTFNQSRYPRILKDSLLPVPKSYDSNIDAWYPPG
NCU02797.1	LLMNSFNTDDDTAAIIKKYEGHNVDILTFNQSRYPRIYKDSLLPVPKSFDSPLHDWYPPG
FG00524.1 	ILMNSFNTNDDTAAIIKKYEGHNVDILTFNQSRYPRVYKDSLLPVPKDNDSPINEWYPPG
AN9148.1  	VLMNSFNTDQDTQSIIKKYQGHNVDIITFNQSRYPRIIKDSLLPAPKSFDAPLQDWYPPG
          	:*******::** :*****:******:*********: ******.**. *: :. *****

MG01631.1 	HGDVFESLYNSGVLDKLIERGIEYIFLSNVDNLGAVVDLRILQHMVETGAEYIMELTNKT
NCU02797.1	HGDVFESLYNSGILDKLIERGIEIIFLSNADNLGAVVDLRILQHMVETDAEYIMELTNKT
FG00524.1 	HGDVFESLYNSGILDKLLERGIEIVFLSNVDNLGAVVDLRILQHMMETDSEYIMELTNKT
AN9148.1  	HGDVFESLYNSGTLDKLLERGVEYIFLSNADNLGAVVDTRILQHMIDTKAEYIMELTDKT
          	************ ****:***:* :****.******** ******::* :*******:**

MG01631.1 	KADVKGGTIIDYEGKVRLLEIAQVPKEHVNEFKSIKKFRYFNTNNIWMNVQAIKRVVENH
NCU02797.1	KADVKGGTIIDYEGSVRLLEIAQVPKEHVNEFKSIKKFKYFNTNNIWMNVQAVKRVVENN
FG00524.1 	KADVKGGTIIDYEGSVRLLEIAQVPKEHVNEFKSIKKFKYFNTNNIWLNLKAIKRVVEND
AN9148.1  	KADVKGGTIIDYEGKVRLLEIAQVPKEHVNEFKSIKKFKYFNTNNIWMNLRAIKRVVEEN
          	**************.***********************:********:*::*:*****:.

MG01631.1 	ELEMEIIPNGKTIPGDKKGESDISIIQLETAVGAAIRHFNNAHGVNVPRRRFLPVKTCSD
NCU02797.1	ELELEIIPNEKTIPGDKKGESDISIVQLETAVGAAIKHFKGAHGVNVPRRRFLPVKTCSD
FG00524.1 	ELEMEIIPNGKTIPGDKKGESDISIVQLETAVGAAIRHFNNAHGVNVPRRRFLPVKTCSD
AN9148.1  	ELEMEIIANEKSIPADKKGEADQAIYQLETAVGAAIRHFKNAHGVNVPRRRFLPVKTCSD
          	***:***.* *:**.*****:* :* **********:**:.*******************

MG01631.1 	LMLVKSDLYTVKHGQLQMSAARFGDAPLIKLGTDFKKVSDFQKRISSIPKMIELDHLTIT
NCU02797.1	LMLVKSDLYTVKHGQLQMSSARFGDAPLIKLGNDFKKVSDFQKRIPSIPKIIELDHLTIT
FG00524.1 	LMLVKSDLYTLKHGQLQMSAARFGDAPLIKLGGDFKKVSDFQKRIPSIPKVLELDHLTIT
AN9148.1  	LLLVKSDLYRLEHGQLVMDPNRFGGVPVIKLGSDFKKVSDFQKRIPSIPRIVELDHLTIT
          	*:******* ::**** *.. ***..*:**** ************.***:::********

MG01631.1 	GAVNLGRGVTFKGTVIIVATEGQTIDIPPGSILENVVVQGSLRLLEH
NCU02797.1	GAVNLGRGVTLKGTVIIVATEGQTIDIPPGSILENVVVQGSLRLLEH
FG00524.1 	GAVNLGRGVTLKGTVIIVATEGSTIDVPPGSILENVVVQGSLRLLEH
AN9148.1  	GAVNLGRNVTLKGTVIIVATEGSTIDIPPGSVLENCVVQGSLRILEH
          	*******.**:***********.***:****:*** *******:***
```
